# 高重现毛细管电泳的研究进展

**DOI:** 10.3724/SP.J.1123.2023.12004

**Published:** 2024-06-08

**Authors:** Zhenpeng GUO, Yi CHEN

**Affiliations:** 1.淮阴工学院矿盐资源深度利用技术国家地方联合工程研究中心, 江苏 淮安 223003; 1. National & Local Joint Engineering Research Center for Mineral Salt Deep Utilization, Huaiyin Institute of Technology, Huai’an 223003, China; 2.中国科学院化学研究所, 中国科学院活体分析化学重点实验室, 北京 100190; 2. CAS Key Laboratory of Analytical Chemistry for Living Biosystems, Institute of Chemistry, Chinese Academy of Sciences, Beijing 100190, China; 3.中国科学院大学, 北京 100049; 3. University of Chinese Academy of Sciences, Beijing 100049, China

**Keywords:** 毛细管电泳, 电泳谱图转换, 非时间测量模式, 高重现谱图, capillary electrophoresis, conversion of electropherograms, non-time measurement mode, highly reproducible electropherograms

## Abstract

毛细管电泳(CE)因具有微量、快速、高效、分离模式丰富等特点得以快速发展并逐渐走向成熟,但在其推广应用过程中一直伴随着出峰稳定性或重现性不佳的问题。CE长期采用信号强度对迁移时间作图的测量模式,但迁移时间并非自变量,受诸多直接和间接因素的影响,故很难测得稳定或精密的电泳谱图。为解决此类问题,国内外很早就开展了不同层次的研究,出现了至少三类解决策略:一是设法控制和稳定电泳特别是影响电渗的条件,以提高出峰的重复性;二是设法调整电泳峰参数,主要是利用内标来校正出峰位置,以提高出峰的重现性,如作时间比例谱、校正时间谱、有效淌度谱、校正淌度谱等;三是寻找建立高重现CE(HRCE)实时测量的新理论、新原理、新方法,从根本上彻底解决问题,如本团队提出的加权淌度谱、迁移电量谱、电密度谱、偏摩尔电密度谱及其比例谱等,这些新式CE方法在适当范围内可以抵抗CE条件或参数的波动,给出高重现的电泳谱图。本综述旨在总结构建HRCE的理论表述和研究进展,阐明影响CE重现性的一些关键因素,核心是电泳峰的表述方式。综述简要归纳分析了CE发展以来文献中对电泳峰表述方式的研究,但不直接涉及和讨论通过仪器改进、实用方法相关的参数的优化、改善等提升CE重复性或重现性的研究及其进展。

## Abstract (en)

Following rapid developments in capillary electrophoresis (CE), this technology has become an established analytical technique owing to its microscale characteristics, high speed, high efficiency, and versatility. However, the challenges of poor peak stability and/or reproducibility have consistently hindered its wider applications. CE has long been used as a measurement tool for plotting signal intensities versus the migration time; however, the migration time is not an independent variable in CE, but is affected by many direct and indirect parameters, including capillary (length, diameter, and inner surface properties), electric field (or voltage, current, and/or power), temperature, and running buffer (electrolytes, additives, solvents, and their concentration, buffering pH, etc.). These intricacies render the acquisition of reproducible electropherograms difficult. Various studies ranging from those on the early stages of CE development to those on the exploration of three important strategies have been conducted to address this issue. In the first strategy, the CE conditions, especially those parameters that can maintain a stable electro-osmotic flow, are strictly controlled and stabilized to significantly improve peak repeatability. In the second strategy, either the peak position is corrected using internal standards or the peak time is converted into other variables, such as electrophoretic mobility, to offset or eliminate some unstable factors, thereby improving the repeatability and even reproducibility of the peaks; this strategy is useful when plotting signals versus the migration time ratio, correlated migration time, effective mobility, or temperature-correlated mobility. In the third strategy, a new methodology called highly reproducible CE (HRCE) is established using theoretical studies to explore better principles for real-time CE with the aim of the complete removal of the challenge from the root. This strategy includes the development of novel methods that plot electropherograms based on weighted mobility, migrated charge, charge density, or partial differential molar charge density. Similar to ordinary CE approaches, this strategy can also draw electropherograms based on the ratios of these properties. As theoretically predicted, these novel methods can offset or resist changes in critical CE conditions (mainly electric field strength, capillary length and diameter, and/or some buffer parameters such as concentration). Our experimental results demonstrate that given certain prerequisites, a new set of methods can produce highly reproducible electropherograms. This review focuses on the theoretical basis and advancements of HRCE, and elucidates the link between electrophoretic migration/peak expression theories and their impact on reproducibility. Studies on the transformation of time-scale electropherograms in the CE literature are summarized and analyzed in general. However, this review does not directly discuss research on and progress in improving CE repeatability or reproducibility through instrument upgrades, parameter optimization, or practical method refinements.

毛细管电泳(CE)因具有高效、快速、微量、通用、绿色、高自动化和能阵列化的特点在20世纪80年代和90年代得以快速发展,并诞生出了多种分离模式,在生命、医药、环境及食品等诸多学科或领域中获得应用。CE的分析对象广泛,从无机离子、有机小分子到生物大分子乃至纳米、细胞等颗粒物。CE也是率先出现的纳升级分离分析新技术,展现出了经济安全、绿色环保等优越性。但在CE走向成熟,并由实验室研究转移至生产过程监测、品质控制、标准化检测等实际应用的过程中,一直面临着重复性或重现性不佳的问题^[1⇓⇓-4]^。重复性对应于精度,以日内、日间分析结果的相对标准偏差考量;重现性则是实验室间参数改变情况下的分析结果对比。以迁移时间为变量的CE谱图和由此获得的分析结果受进样、毛细管、温度、电驱动力、缓冲液等诸多实验参数和因素的影响,它们的改变导致CE出峰的不稳定和不重复。仪器系统、材料、环境等的差异和难以调控的变化,给CE重现性和方法转移造成了困难。

如何得到稳定、重现的CE结果成为CE发展过程中的重大挑战,后续相继出现了不同角度、不同层面的研究,归纳起来大致有三大类:第一类是严密控制CE的各种条件;第二类是峰的校正技术;第三类是重建CE测量方案,构建高重现毛细管电泳(HRCE)谱图和方法。本综述在简要回顾前两类研究进展的基础上,着重分析HRCE研究进展,试图从基本理论出发,阐明影响CE重现性的一些关键因素;并且通过提炼相关研究的本质特点,揭示出未来值得重点研究的方向。本综述不直接涉及精密度即重复性相关的参数控制(如仪器、方法发展参数的优化、改善等研究及其进展等)。

## 1 CE条件与控制

### 1.1 基本理论

传统CE测量区带信号随时间变化,给出以时间为自变量的电泳谱图,可简记为*t*-谱,其描述源自速度理论。考虑样品出峰时间为*t*,毛细管进样口到达检测窗口的长度为*L*_d_,则样品的峰值迁移速度(*υ*)为^[5⇓⇓-8]^:


(1) $$\left\{\begin{array}{l} \upsilon =\frac{L_{d}}{t}=\mu E=\mu \frac{V}{L}\\ t=\frac{L_{d}}{\upsilon }=\frac{L_{d}}{\mu E}=\frac{L_{d}L}{\mu V} \end{array}\right.$$

式中,*E*为电场强度,是施加于毛细管两端电压*V*与毛细管总长*L*之比;*μ*为样品的单位电场速度,原定义为淌度,但CE中实际测得的是权和淌度,因为它是电泳(ep)、电渗(os)和相分配(*k*)的共同贡献,实为加权之和:


(2) $$\left\{\begin{array}{l} \mu =\frac{\upsilon }{E}=\upsilon \frac{L}{V}=\frac{n_{b}}{n_{b}+n_{s}}\mu_{b}+\frac{n_{s}}{n_{b}+n_{s}}\mu_{s}\\ =\frac{1}{1+k}(\mu_{os}+\mu_{ep})+\frac{k}{1+k}(\mu_{os}+\mu_{s,ep})\\ =\mu_{os}+\frac{\mu_{ep}+k\mu_{s,ep}}{1+k}\\ k=\frac{n_{s}}{n_{b}} \end{array}\right.$$

式中,*n*是样品分子的个数或物质的量,*k*是(纯色谱中的)保留因子;b是电泳缓冲液;s是(准)固定相。注意, *μ*_ep_是在实际溶液中观测到的有效淌度,是各级离子的加权和:


(3) $$\mu_{ep}=\sum_{j}\alpha_{j}\;\mu_{j,ep}^{0}$$

式中,*α_j_*为目标分子的第*j*级解离度或其他平衡决定的解离度, $\mu_{j,ep}^{0}$ 为第*j*级离子的绝对淌度(外推到零浓度的值)。在较稀溶液中,离子的*μ*_ep_也可用Henry公式^[9]^计算:


(4) $$\left\{\begin{array}{l} \mu_{ep}=\frac{\varepsilon \zeta f(r/d)}{\eta }=\left\{\begin{array}{l} \frac{\varepsilon \zeta }{\eta },\;atr/d\to \infty \\ \frac{2\varepsilon \zeta }{3\eta },\;atr/d\to 0 \end{array}\right.\\ d≌\sqrt{\frac{\varepsilon RT}{2cF^{2}}} \end{array}\right.$$

式中,*ε*和*η*是缓冲液的介电常数和黏度,*ζ*为切变界面上的电动电位,*d*是双电层厚度,*r*是电动粒子的溶剂化半径,*R*为气体普适常数,*T*为热力学温度,*c*为离子浓度,*F*为法拉第常数。依据Ohshima估算^[10,11]^, *f*(*r/d*)取值介于1(*r/d*→∞)~2/3(*r/d*→0)。考虑到电渗源于毛细管壁和(准)固定相上的电荷,而(准)固定相粒径通常较大,它和毛细管的*r/d*→∞,则*μ*_os_=*ε*(*ζ*_cap_+*ζ*_s_)/*η*=*εζ*_os_/*η*(其中*ζ*_cap_和*ζ*_s_分别是毛细管内壁和(准)固定相的电动电位)。考虑到样品溶液较稀,合并公式(1)~(4),整理得:


(5) $$\begin{array}{cc} t & =\frac{LL_{d}}{V}\frac{1+k}{\sum_{j}\alpha_{j}\mu_{ep}^{0}+k\mu_{s,ep}+(1+k)\mu_{os}}\\ & ≅\frac{\eta LL_{d}}{\varepsilon V}\frac{3(1+k)}{2\sum_{j}\alpha_{j}\zeta_{j}^{0}+3k\zeta_{s}+3(1+k)\zeta_{os}} \end{array}$$

式中, ${\zeta_{j}}^{0}$ 是样品第*j*级离子的无限稀释或极限电动电位。公式(5)第二式针对粒径小的离子样品,即*f*(*r/d*)≈2/3。公式(5)已基本揭示了毛细管内分离所涉及的因素,但从仪器测试角度考虑,CE还包括进样和检测两个部分,用样品区带的展宽贡献或总体分离效率计算,应有以下公式^[5,6]^:


(6) $$\left\{\begin{array}{l} N=\frac{L_{\text{d}}^{2}}{\sigma^{2}}\\ \sigma^{2}=\sigma_{\text{inj}}^{2}+\sigma_{\text{sep}}^{2}+\sigma_{\text{d}}^{2}\\ \;=\underset{色谱}{\underset{︸}{\sigma_{\text{LD}}^{\text{2}}+\sigma_{\text{RD}}^{\text{2}}+\sigma_{kF}^{2}}}+\underset{电泳}{\underset{︸}{\sigma_{\text{Ed}}^{\text{2}}}}+\underset{通项}{\underset{︸}{\sigma_{T}^{2}+\sigma_{\text{inj}}^{2}+\sigma_{\text{d}}^{2}}}\\ \;≅A_{1}2Dt+A_{2}d_{s}{\left(\frac{\mu d_{s}}{D}\right)}^{1/3}+A_{3}\frac{\mu d_{s}}{D}+\\ \;f\frac{\mu_{\text{ep}}}{\mu_{\text{b,ep}}}+f(\Delta T)+\frac{L_{\text{inj}}^{2}}{12}+\frac{{\left(vt_{\text{rsp}}\right)}^{2}}{12}\\ \Delta T=\frac{Q_{\text{w}}r_{\text{cap}}^{\text{2}}}{2}\left(\frac{1}{2\kappa_{1}}+\frac{1}{\kappa_{2}}\ln\frac{r_{\text{cap,2}}}{r_{\text{cap,1}}}+\frac{1}{\kappa_{3}}\ln\frac{r_{\text{cap,3}}}{r_{\text{cap,2}}}\right.\\ \left.\;\frac{1}{q_{\text{w}}r_{\text{cap,3}}}\right)\Rightarrow \Delta T_{\max}=\frac{Q_{\text{w}}r_{\text{cap}}^{\text{2}}}{4\kappa_{1}}\\ Q_{\text{w}}=2E^{2}c\Lambda r_{\text{cap}} \end{array}\right.$$

式中,下标inj、sep、d、LD、RD、*kF*、*T*、Ed分别指进样、分离、检测、轴向扩散、径向或涡流扩散、传质阻力、温度、电场(畸变)加宽因素,下标0、1、2、3分别表示管中心、管内壁、管外壁、聚酰亚胺外表面,*A*_1_、*A*_2_、*A*_3_为常数,*μ*_b,ep_为缓冲试剂离子有效淌度,*d*_s_为(准)固定相粒径,*L*_inj_为进样区带长度,*t*_rsp_为检测系统总响应时间,*D*为扩散系数,Δ*T*为毛细管径向温度差,*r*_cap_为毛细管半径,*κ*为热导率,*q*_w_为热流速率,*Q*_w_为单位体积电功率,*Λ*为电泳介质的摩尔电导。

### 1.2 影响因素与类型

由公式(5)与(6)可知,CE效率与进样、检测、*t*等一系列因素有关,而*t*并非自变量而是因变量,是多种直接和间接因素的函数。归纳起来,CE有直接和间接因素两大类。

直接因素包括进样参数(*L*_inj_=*υt*_inj_,电进样*υ*=*μE*,压力进样 $\upsilon =r_{cap}^{2}\Delta P/8L\eta$ )、毛细管参数(*L*、*L*_d_、*r*_cap_)、毛细管温度*T*、电驱动力(*V*、*E*、*I*(电流)、*W*(功率),有时候会施加气压差Δ*P*)、缓冲液参数(试剂与添加剂种类、*c*、pH等)、检测器响应时间和数字化相关的采样频率等。它们都是可调、可优化或可控制的,有些可以通过仪器化、自动化获得精密控制。但值得注意的是,低浓度或pH≠p*K_a_*±1的电解质溶液没有pH缓冲能力。

间接可调因素有*α*、*ε*、*η*、*κ*、*μ*、*ζ*、*d*、*k*、*q*_w_、*r*、*t*、*D*、*Q*_w_、Δ*T*等,它们既受直接参数的影响(特别是*T*、*c*、pH、缓冲液与(准)固定相性质等),又受间接因素影响,很难通过直接因素的精准调控获得稳定。这些间接因素可能互为函数,难以优化和进行精准、稳妥的调控。经过一段时间的研究,发现设法稳定*μ*特别是*μ*_os_至关重要,因为*μ*_os_值远大于*μ*_ep_。

### 1.3 关键因素

在CE发展不久,多数研究者就意识到需要优化和严格控制直接因素,这能在很大程度上改善出峰的重复性(精密度),但由于间接因素(至少部分)会随仪器系统、材料、环境等变化,即使直接因素获得了稳定控制,间接因素也会对重现性检验和方法转移等造成困难。已知会影响CE结果的因素有:不同仪器间的进样、毛细管卡盒、控温、检测等存在的材料和设计差异,毛细管尺寸因用料和制作工艺不同造成的批次(乃至同批次不同部位)差异,毛细管因活化及冲洗方法不同造成的内壁表面物性差异;另外,因试剂、实验室装备、人员等的不同,采用的缓冲液会存在浓度、pH波动,有时候配制方法也不尽相同。这给CE方法的检验和转移造成了困难。研究认为,为提高精密度或更容易转移CE的方法与结果,必须高度重视CE分离的各种细节^[12⇓⇓⇓⇓-17]^。下面仅对若干容易忽视的条件和因素进行概述。

#### 1.3.1 毛细管

除精确控制*L*和*L*_d_外,要注意*r*_cap_的选择。已知*r*_cap_越小,分离效率越高,但检测光路也越短,需在效率与灵敏度间折中选择。多年实践表明,选用30~50 μm内径的毛细管是适宜的。另外还要注意毛细管切口的平整度^[18,19]^。

毛细管内壁性状控制十分重要,它影响*k*、非特异性吸附、电渗大小和方向。必须在进样前充分洗涤(或活化)管壁并与缓冲液充分平衡^[20,21]^。利用涂层技术改性管壁一直是CE研究的重要课题。涂层除能调控和稳定电渗、抑制非特异性吸附外,还可引入或强化相分配机制。目前有物理吸附和化学键合涂层制备方法,涂层材料有纤维素及其衍生物^[22⇓⇓-25]^、聚乙烯醇、聚环氧乙烷、离子液等^[26,27]^。能调控甚至改变电渗方向的涂层主要是高价阳离子和胺/季铵盐类,如二价金属阳离子、烷基胺、阳离子表面活性剂和阳离子聚合物等,它们可通过静电吸附到管壁上,改变管壁的电荷性质和多寡,进而改变电渗方向和强弱^[28,29]^。此外也有一些关于纳米涂层的研究报道,旨在通过增加比表面积来提升分离能力^[30⇓⇓⇓-34]^。需要注意的是,改性涂层都有各自的适用范围和限制条件,恰当条件(如缓冲液pH、离子强度、冲洗方法等)会促进分离,而不当的条件则会破坏涂层,加剧不重现的问题。另外,涂层管批次间的重现性也依然是一个挑战。

#### 1.3.2 缓冲溶液

电泳缓冲液是极其重要但又是最难调控的CE条件。它直接决定介电常数和黏度,不仅影响管壁状态及其电荷进而影响电渗,还直接决定样品的解离度和保留因子。缓冲液分溶剂和溶质两部分。溶剂决定介电常数、黏度值,影响电渗大小和样品溶解度。溶质包括缓冲试剂(弱电解质)和功能性添加剂两大部分,电解质决定电流、发热量、pH值等;添加剂可调控黏度、电渗、分离机制等。缓冲液极小的变动可能会引起出峰时间极大的改变^[35]^,故而在优化和配制缓冲液时需要非常仔细,特别是缓冲液组成、浓度、pH值等^[36⇓-38]^。理想的缓冲体系pH缓冲容量高、光吸收弱或无光吸收、溶剂蒸发可忽略、组成浓度恒定不变。

缓冲液电解质的离子类型及浓度对电渗流(EOF)、样品淌度、分离效率等的影响,以及通过调节缓冲液溶剂组成、添加剂提升CE分析精密度的研究,可参考相关文献[39⇓⇓⇓⇓⇓-45]。缓冲液pH在<2.5和8.0~10.0范围内,EOF的稳定性比较好,CE出峰的重复性较高^[46,47]^。Strieglerová等^[48]^用2.5 mol/L乙酸(pH 2.0)分离17种氨基酸,迁移时间的相对标准偏差(RSD)为0.14%~0.28%(*n*=7)。Guo等^[49]^采用同浓度乙酸,在不控温条件下分离37种离子、生物胺和氨基酸,迁移时间的日内、日间RSD均<1.95%。

#### 1.3.3 温度

CE过程中产生的焦耳热会改变温度,增大管内温度梯度,影响缓冲溶液、样品及毛细管壁的理化性质,改变迁移速度。温度对缓冲液的黏度*η*影响大,在20~40 ℃范围内,*η*的温度系数约为-2%/℃^[50]^,因*ζ*、*ε*随温度的变化小^[51]^,所以迁移速度的温度变化系数主要由*η*决定,即+2%/℃。持续或骤然产生的焦耳热还可能造成溶剂蒸发和气泡产生,进而阻断电泳。气泡会导致局部放电,可击裂管壁、破坏缓冲液或样品成分等。可见,焦耳热造成的毛细管内温度变化是影响CE分析精密度的关键因素,所以在CE发展早期就获得重视和研究^[52⇓⇓⇓-56]^,通常通过对毛细管主动散热控温、选择低电导率的缓冲液、小内径的毛细管等措施抑制焦耳热。

成熟的商品毛细管电泳仪器都有恒温控制,采用液冷或风冷的方式对毛细管主动散热,其中液冷方式的控温效果更好^[57]^。但多数未对毛细管两端和检测部位恒温,造成毛细管控温部分与两端存在温度差和样品迁移速度差。Krylov等^[58,59]^发展了测算控温和非控温毛细管内部溶液温度的方法,结果表明即使用低电导率的缓冲液,两部分的温差也超过15 ℃,可能造成较大的分析结果误差^[60,61]^。不同仪器的控温方式、仪器外部环境不同等,控温效果存在差异,CE方法转移时需对温度设定进行考察^[62]^。

## 2 峰校正技术

上述讨论的因素控制,一般只能改善分离的重复性或精密度,无法改善出峰的重现性。为此出现了峰校正技术,它以内标作参比,将出峰时间换成比例值、校正值或有效淌度值等,能在一定范围内提升CE谱峰的重复性和重现性。

### 2.1 时间比例谱

以样品与内标的迁移时间之比(*R_t_*)代替*t*为横坐标作CE谱图,得到时间比例谱(*R_t_*谱)。由公式(5)可得:


(7) $$R_{t}=\frac{t}{t_{IS}}=\frac{1+k}{1+k_{IS}}\frac{\sum_{j}\alpha_{IS,j}\mu_{IS,ep}^{0}+k_{IS}\mu_{s,ep}+\left(1+k_{IS}\right)\mu_{os}}{\sum_{j}\alpha_{j}\mu_{ep}^{0}+k\mu_{s,ep}+(1+k)\mu_{os}}≅\frac{1+k}{1+k_{IS}}\frac{2\sum_{j}\alpha_{IS,j}\zeta_{IS,j}^{0}+3k_{IS}\zeta_{s}+3\left(1+k_{IS}\right)\zeta_{os}}{2\sum_{j}\alpha_{j}\zeta_{j}^{0}+3k\zeta_{s}+3(1+k)\zeta_{os}}$$

式中,IS表示内标。显然,公式完全消除了*η*、*ε*、*L*、*L*_d_和*V*的影响;若内标峰位置靠近样品,则式中的分子、分母各项的变化也非常接近,大致可以互相抵消,给出相当重现的结果。当选择电渗为参考内标时,分子中因*k*_IS_=*ζ*_IS_=0,只剩下*ζ*_os_一项。此时*R_t_*峰的重现性取决于各电动电位与*ζ*_os_的比值和保留因子,若它们稳定,则*R_t_*峰稳定。Yang等^[63]^在2天内对6种氨基酸样品进行了60次分离,结果显示,*R_t_*峰的RSD从原来*t*峰的2.1%~4.6%改善到0.6%~1.1%,还能减小电压(14~22 kV)、温度(16~44 ℃)、毛细管尺寸(50、75 μm)、环糊精添加剂对出峰的影响。该法得到了较多的应用^[64⇓⇓-67]^。Zheng等^[68]^将*R_t_*技术用于阵列毛细管电泳成像分析(图1),实现了鼠脑切片中不同氨基酸类神经递质的成像研究。

**图 1:**
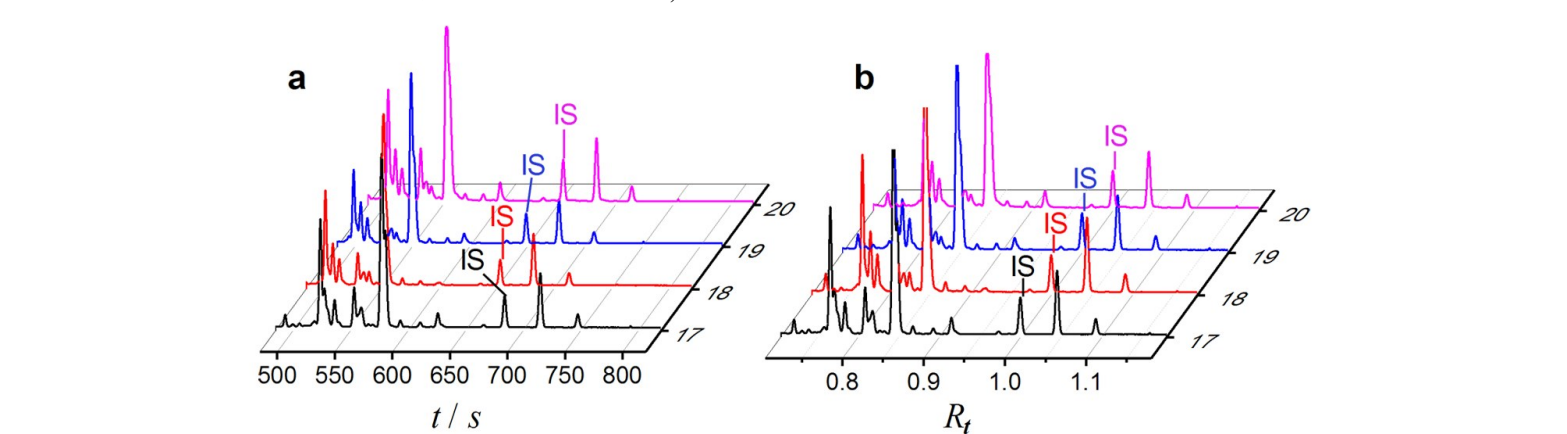
鼠脑切片样品的(a)迁移时间谱图与(b)迁移时间比谱图^[68]^

注意,式(7)成立的前提是,缓冲液黏度与样品和内标没有相互影响或可忽略,且平均温度在检出样品和内标物时不变。这要求内标和样品的出峰时间越近越好,距离太远则差别过大,*R_t_*效果不好甚至可能更差。为获得较好的结果,需要重新选择内标。

### 2.2 校正时间谱

内标峰也可用于校正电渗或样品出峰时间,以减小乃至消除电渗波动的影响。其做法是向目标组分标准样品(ss)和实际样品(rs)中分别加入IS,并分别进行CE分析。假设目标组分(*i*)的迁移速度在ss和rs中保持不变,则可由IS峰计算出ss与rs间的电渗差Δ*μ*_os_,见式(8):


(8) $$\Delta \mu_{\text{os}}=\mu_{\text{rs},i}-\mu_{\text{ss},i}=\mu_{\text{rs},\text{IS}}-\mu_{\text{ss,IS}}=\frac{LL_{d}}{V}\left(\frac{1}{t_{\text{rs},\text{IS}}}-\frac{1}{t_{\text{ss,IS}}}\right)$$

从rs的淌度中扣减Δ*μ*_os_,得到式(9):


(9) $$\mu_{\text{ss},i}=\mu_{\text{rs},i}-\Delta \mu_{\text{os}}=\frac{LL_{d}}{V}\left[\frac{1}{t_{\text{rs},i}}-\left(\frac{1}{t_{\text{rs},\text{IS}}}-\frac{1}{t_{\text{ss,IS}}}\right)\right]$$

由此可将*t*_rs,_*_i_*校正为标准样品的 $t_{ss,i}$ ^[69]^:


(10) $$\frac{1}{t_{ss,i}}=\frac{1}{t_{rs,i}}-\frac{1}{t_{rs,IS}}+\frac{1}{t_{ss,IS}}$$

此方法用于校正与内标迁移时间相近的组分,能提高峰位置的重复性。用19种衍生氨基酸进行测定,结果表明:与内标邻近的组分,其单内标校正的日内校正出峰时间的RSD可从1%减小到0.5%,但离内标较远者则增加到0.5%~1.3%^[69]^。在此种情况下,需要更换单内标,或使用双内标来同时校正组分淌度和电渗^[69⇓-71]^。

同样采用上述氨基酸样品,在20天内进行10次CE分析,其出峰时间的RSD为3%~10%,经单内标校正后改善到1%左右,而经双内标校正则改善至0.5%^[69]^。显然,双或多内标校正优于单内标校正。该方法的问题是校正操作需要事后进行,计算也比较复杂;对于复杂样品,也难以找到不与样品峰重叠的内标,故其推广难度颇大。

### 2.3 有效淌度法

将CE出峰时间扣除电渗,还原为有效淌度,这是一种接近经典电泳的校正法。已知有效淌度*μ*_ep_主要由样品及其所在缓冲溶液的物化性质决定,与电渗、电压或电场强度无关,也不包括毛细管尺寸等相关参数,因此能提高测量的精度和重现性。Schmitt-Kopplin等^[72,73]^用3种代表性样品作了76次CE分析,结果表明:出峰时间的RSD为1.02%~4.63%,而*μ*_ep_的RSD为0.60%~1.48%,明显优于前者。*μ*_ep_值在12~30 kV之间有较强的抑制电压变化的能力。

有效淌度法推动了CE-MS在代谢组学中的研究和应用^[74]^。因*μ*_ep_有较强的特征性,故在一定条件下可结合CE-MS鉴定代谢成分。通过将*t*谱图转换为*μ*_ep_谱图,Drouin等^[75]^发展了可检测450种内源性酸性和碱性代谢物的CE-MS方法,并建立了相关的*μ*_ep_实验数据库,可用于培养人细胞中代谢物的分析。他们还组织了11个国家共13个实验室的16个CE-MS平台,对其所建方法进行了验证,使用10%(v/v)乙酸缓冲溶液,要求各实验室用相同的方法配制,但其他实验参数(如毛细管、接口、进样量、电压、温度、毛细管平衡与冲洗等)由实验室自己酌情决定。结果表明,在实际样品(水、血浆、尿液)中添加20种模型化合物,其*μ*_ep_的RSD≤3.1%,优于*R_t_*谱的10.9%^[76]^。Gonzalez-Ruiz等^[77⇓-79]^还开发了ROMANCE(Robust Metabolomic Analysis with Normalized CE)软件,方便将CE-MS数据由*t*-值转换为*μ*_ep_-值。该软件兼容代谢组学分析软件。

需要注意的是,*μ*_ep_受离子强度、pH、黏度、温度、焦耳热或电场强度等影响,且CE中的*μ*_ep_主要由权和淌度扣减*μ*_os_得到,这会受电渗波动的影响。为减少此类影响,宜采用准确的管内液体流速实时测量值来计算。

目前已发展了若干测量毛细管内液体流速的方法。第一种是激光漂白测量法。利用激光诱导荧光装置,当缓冲液中存在荧光试剂如罗丹明B等时,可在检测窗口前一定位置用激光照射,漂白荧光物质并在检测窗口检出,进而计算出管内液流速度^[80,81]^。激光漂白测量法可反复实施,随时测定流速,借此可求算多次测量的平均流速。

第二种是热标记测量法。它在检测窗口前一定位置上,对约0.5 mm长的毛细管施加数十到数百ms的热脉冲,使内部缓冲液因受热而产生浓差区带。此区带被电渗带经检测窗口,由非接触电导检出,可直接计算电渗速度^[82,83]^。

第三种是流量计测量法。Kehl等^[84]^将一耐高电压的流量传感器与毛细管串联,能实时在线测量分析窗口内的平均流速*υ*_q_,用以计算组分的净迁移速度,*υ_i_*=*υ*_det,_*_i_*-*υ*_q_,式中*υ*_det,_*_i_*是CE的出峰速度。用荧光素检验的结果如下:9次*υ_i_*峰的RSD为0.14%,远优于*t*峰的3.54%,亦优于中性IS校正峰的0.59%。在测定荧光标记氨基酸时,*υ_i_*峰的RSD为0.44%,也远胜于*t*峰的3.32%。与前两种方法不同,该方法不干扰CE过程,还能测量管内因重力、压差或其他流体力学原因引起的流动,无需内标或其他技术帮助。

### 2.4 温度校正淌度

Petersen等^[85⇓-87]^通过测定零功率时毛细管内电阻及监测分离过程中的电流变化,利用扣除电流波动来抵消焦耳热效应,发展了温度校正淌度法*μ*(*T*)。在电场强度、温度变化条件下,所得*μ*(*T*)的重现性明显优于*t*峰及未校正的*μ*。

## 3 重建CE测量方案

上述讨论表明,无论是严格控制CE条件,还是校正出峰参数,均较繁琐,都未能彻底解决CE重现性的问题。为此,本实验室^[5⇓⇓-8,88⇓⇓-91]^提出了重建CE测量方案的思路,通过对电动分离理论进行梳理和统一表述,推导出了系列CE实时测量或谱图表述的新方案,其中包括HRCE方法,并进行了实验验证。

考虑CE过程存在微变,即:


(11) $$\upsilon =\mu E=\frac{dl}{dt}$$

式中,*l*为迁移距离。

结合校正欧姆定律*I*=*σES*(*σ*为缓冲液电导, $S=\pi r_{cap}^{2}$ 为毛细孔横截面积),得:


(12) $$\mu E=\mu \frac{I}{S\sigma }=\frac{dl}{dt}$$

或


(13) $$\int_{0}^{L_{d}}\sigma Sdl=\;\int_{0}^{t}\mu Idt$$

考虑同一分离,假设*σ*沿轴线的变化和*μ*随时间的变化皆可忽略,积分得到:


(14) $$q=\int_{0}^{t}Idt=\;\frac{\sigma }{\mu }\int_{0}^{L_{d}}Sdl=\;\frac{\sigma V_{d}}{\mu }≌\frac{\sigma }{\mu }\pi \;r_{cap}^{2}L_{d}$$

式中,*V*_d_为迁移体积,*q*为*t*时间内迁移的电量,它们都是可测量的量,其中*q*可实时测量。式中的电导率可由Kohlrausch定律及Stokes-Einstein、Nernst-Einstein方程^[92⇓-94]^转换成缓冲电解质的淌度:


(15) $$\left\{\begin{array}{l} \lambda_{j}=\frac{z_{j}eF}{6\pi r_{j}\eta }\\ \sigma =\sum_{j}c_{j}z_{j}\lambda_{j}=c_{ebf}F(\mu_{b+}^{0}+\mu_{b-}^{0})\\ c_{ebf}=\alpha \gamma cz,\;atz=z^{+}=|z^{-}| \end{array}\right.$$

式中,*c_j_*、*z_j_*、*λ_j_*分别为第*j*种离子的浓度、价数、等效电导率,*e*为电子电量(取绝对值), *F*为法拉第常数,*r_j_*为离子半径,*α*是解离度,*γ*为活度系数,b表示缓冲液,正负号指示正负离子,*c*_ebf_是缓冲液中电解质折合成+1和-1价后的活度。

将式(15)代入(14)得:


(16) $$q=\int_{0}^{t}Idt=\;\frac{\sigma V_{d}}{\mu }≌c_{ebf}FV_{d}\;\frac{\mu_{b+}^{0}+\mu_{b-}^{0}}{\mu }$$

因为*I*与时间的积分可以实时测量,故用*q*可代替*t*作实时CE,所得电泳谱图可称为迁移电量或电量谱,记作*q*-谱,对应新式CE称为*q*-CE。公式(16)显示,*q*-CE不受电场(或*I*、*W*)影响,只受缓冲液浓度、毛细管参数和淌度比的影响。淌度比抵消了*ε*、*η*的影响,所以*q*-谱有较强的抗温变能力,这也有利于提高*q*峰的精密度。

注意,*q*峰随毛细管参数变化,为此,可将公式(16)除去*V*_d_,求得电密度(*ρ*):


(17) $$\rho =\frac{q}{V_{d}}=\frac{\sigma }{\mu }≌c_{ebf}F\;\frac{\mu_{b+}^{0}+\mu_{b-}^{0}}{\mu }$$

显然以*ρ*代替*q*或*t*可构建重现性更好的*ρ*-CE新方法,输出的*ρ*峰位置与毛细管尺寸、*E*(*I*、*W*)无关。我们实验室的比较研究^[89]^表明,*t*-谱随电压、管径、管长变化(图2a),但*ρ*-谱不变,是高重现的(图2b)。在毛细管不恒温条件下,*ρ*峰位置的RSD还能保持在0.97%~1.02%范围内。进一步研究表明,当毛细管恒温温度从20 ℃提升至30 ℃时,4种组分的*t*峰位置变化为-1.34%/℃~-1.41%/℃(图3a),与电流增幅平行(图3b);而*ρ*峰位置变化幅度为-0.65%/℃~-0.73%/℃,亦即*ρ*-谱的抗温变能力比*t*-谱高不止一倍。Lee等^[95]^曾报道迁移指数法,他们考虑了电流密度积分,但未考虑*L*与*t*间的关系,所以效果不如*ρ*-谱。

**图 2:**
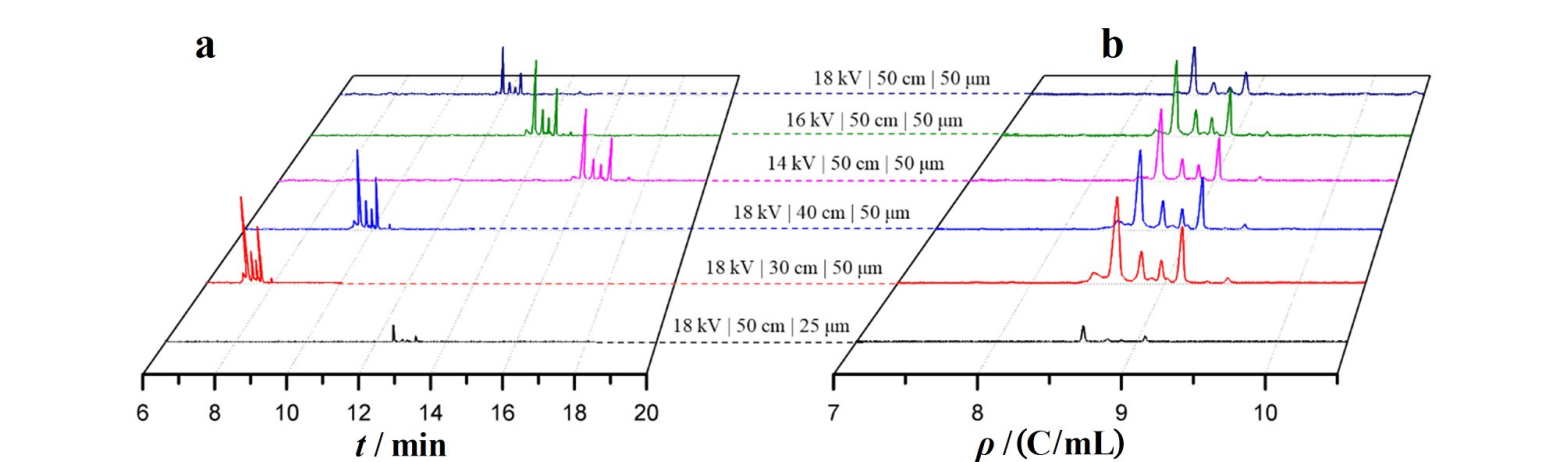
分离电压、毛细管有效长度与管径对(a)迁移时间谱与(b)电密度谱的影响^[89]^

**图 3:**
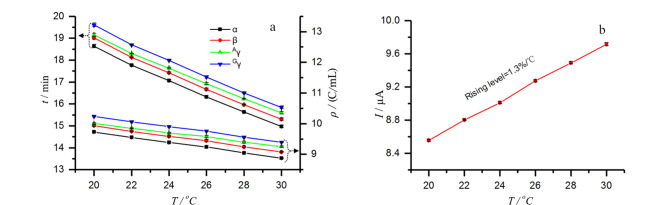
温度对(a)迁移时间、电密度和(b)电流的影响^[89]^

理论上,还可以进一步从*ρ*消去缓冲液浓度*c*项,排除其影响。但实测表明,简单的扣除*c*项不能得到预期结果。事实上,缓冲液浓度与电解质价态、解离度、添加剂乃至溶剂等因素有关,不能像电场等一样直接去除,须在固定某些条件下,进行偏微分操作,比如可对缓冲试剂、某一离子甚至某一添加剂浓度做偏微分,得到的偏摩尔电密度,记为*g*:


(18) $$g=\frac{\partial \rho_{R}}{\partial c_{bi}}\approx \frac{\rho_{R}}{c_{ebf}}=F\;\frac{\mu_{b+}^{0}+\mu_{b-}^{0}}{\mu }$$

式中,*c*_b_*_i_*是缓冲液中某种组分的浓度。由公式(18)可建立*g*-CE方法,作*g*-谱。由图4可见,*g*-谱有一定的抗浓度变化的能力^[4]^。*g*-CE进一步突出了样品*μ*的变化,不仅具备*ρ*-CE的能力,而且还能部分消除缓冲液中某种组分浓度对出峰的影响。对比图5a和5b,在非胶筛分CE分析中,在添加剂聚乙烯吡咯烷酮(PVP)浓度变化的情况下,*g*峰仍有一定的重现性^[69]^。

**图 4:**
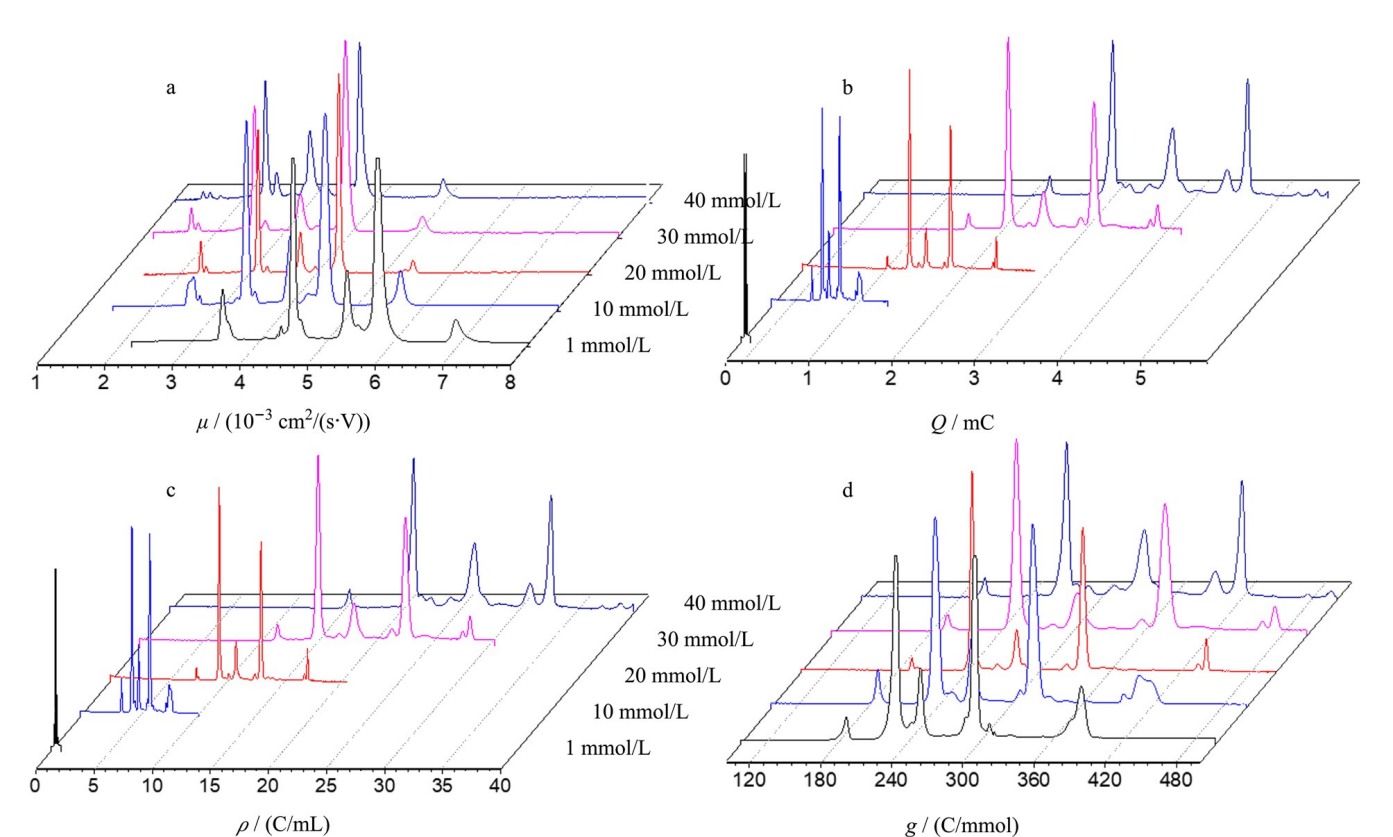
电泳缓冲液浓度对(a)权和淌度谱、(b)迁移电量谱、(c)电密度谱和(d)偏摩尔电密度谱的影响^[8]^

**图 5:**
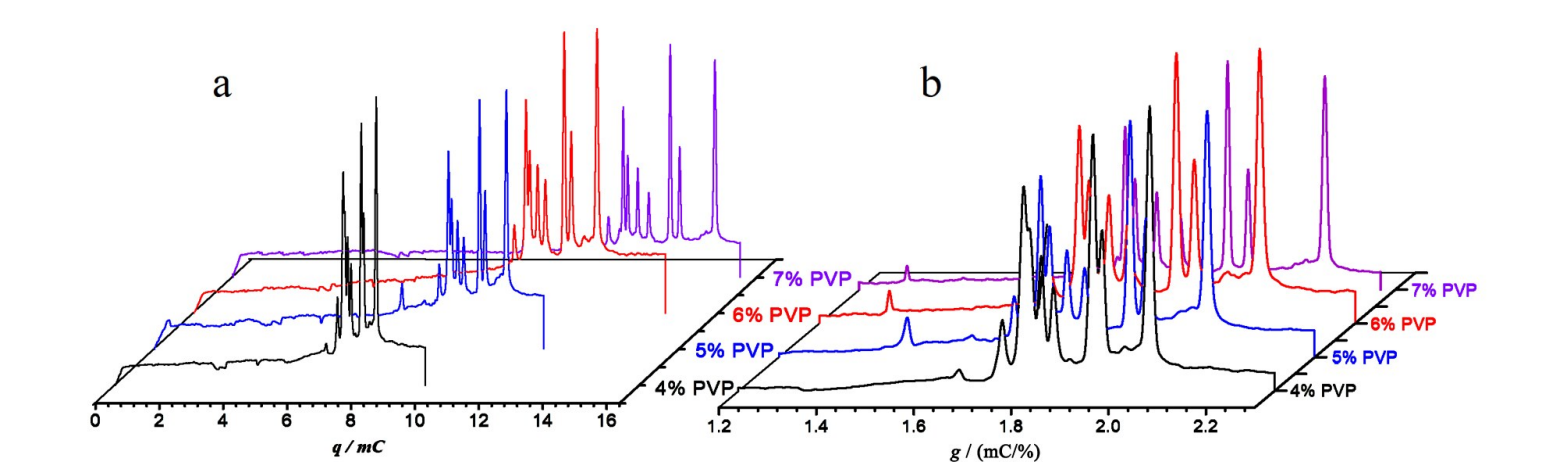
电泳缓冲液中添加剂浓度对(a)迁移电量谱和(b)偏摩尔电密度谱的影响^[90]^

需要注意的是,在进行*ρ*-CE和*g*-CE前,最好事先表征或测算*c*_ebf_、*V*_d_等参数,以便能更可靠地构造相关的谱图。

很显然,除了*q*、*ρ*和*g*外,还可以利用公式(2),直接构建权和淌度谱或*μ*-谱,我们的实验结果显示,它能抵抗电参数对*μ*的影响(图6)。它与校正有效淌度谱相比的优势是,*μ*-谱能实时测量,无须进行复杂的计算。模仿(气相)淌度谱,可以把*μ*-谱称作液相淌度谱。

**图 6:**
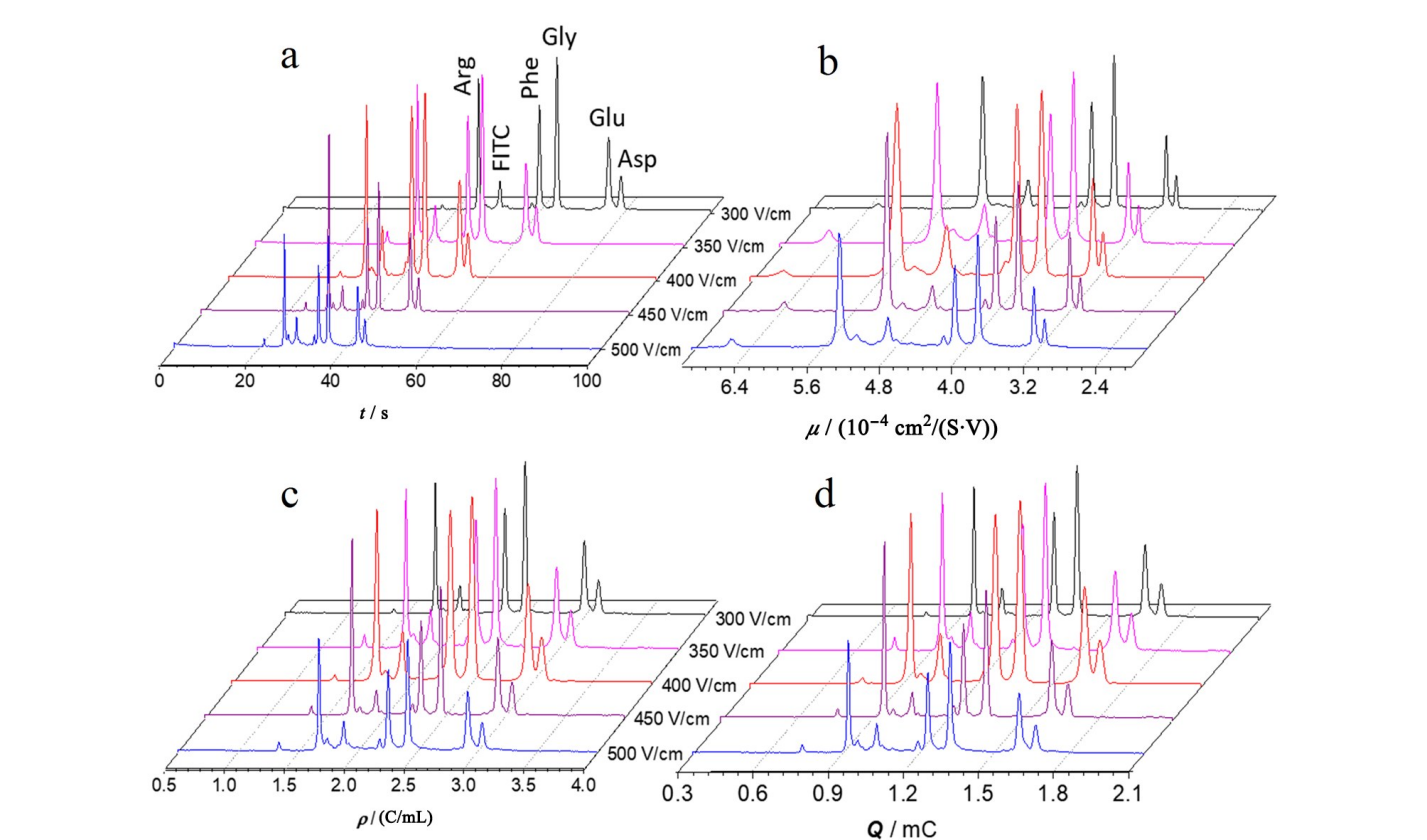
分离电压对(a)迁移时间谱、(b)权和淌度谱、(c)电密度谱和(d)迁移电量谱的影响

总体而言,*q*-、*ρ*-、*g*-谱都能在一定条件下输出高重现谱图,一般不用校正。不过,样品和缓冲液电解质的解离与pH和温度的关系甚为复杂,目前还无法完全从公式中消除,所以前面的谱图校正技术同样也可以使用,以获得更为精密的电泳谱图,其中最简单的校正方法是用内标做比例谱:


(19) $$R_{q}=R_{\rho }=R_{g}=\frac{1}{R_{\mu }}=\frac{(1+k_{IS})\mu_{os}+\mu_{IS,ep}+k_{IS}\mu_{s,ep}}{(1+k)\mu_{os}+\mu_{ep}+k\mu_{s,ep}}$$

若内标为中性分子,内标和目标样品都取点电荷(*f*(*r/d*)=2/3),则有:


(20) $$R_{q}=R_{\rho }=R_{g}=\frac{1}{R_{\mu }}=\frac{\mu_{os}}{\mu_{os}+\mu_{ep}}=\frac{\zeta_{os}}{\zeta_{os}+\zeta_{ep}}$$

如前所述,比例谱不能实时分析,只能实验后换算。

## 4 总结与展望

本文系统地综述了如何获得或构建重复、重现CE的研究进展,推介了*μ*-、*q*-、*ρ*-、*g*-CE,它们具有各自的高重现范围和特点,可能会促进CE的进一步发展,值得推广应用。在没有新的关于*q*-、*ρ*-、*g*-CE商品仪器推出之前,用现有CE商品仪器做*μ*-谱也是可取的,或采用实验后校正技术。总之,研究发展高重现CE方法已经开始并取得了一定进展,但研究并未结束,或许还有其他理论和原理,可用来构建更新、更好的高重现CE方法,期待CE工作者一起努力,推进此类探索和研究。
